# NMR Studies of Tau Protein in Tauopathies

**DOI:** 10.3389/fmolb.2021.761227

**Published:** 2021-11-11

**Authors:** Kristine Kitoka, Rostislav Skrabana, Norbert Gasparik, Jozef Hritz, Kristaps Jaudzems

**Affiliations:** ^1^ Laboratory of Physical Organic Chemistry, Latvian Institute of Organic Synthesis, Riga, Latvia; ^2^ Institute of Neuroimmunology, Slovak Academy of Sciences, Bratislava, Slovakia; ^3^ AXON Neuroscience R&D Services SE, Bratislava, Slovakia; ^4^ Central European Institute of Technology, Masaryk University, Brno, Czech Republic; ^5^ Faculty of Science, National Centre for Biomolecular Research, Masaryk University, Brno, Czech Republic; ^6^ Department of Chemistry, Faculty of Science, Masaryk University, Brno, Czech Republic; ^7^ Faculty of Chemistry, University of Latvia, Riga, Latvia

**Keywords:** tau, nuclear magnetic resonance, protein structure, Alzheimer’s disease, filaments

## Abstract

Tauopathies, including Alzheimer’s disease (AD), are the most troublesome of all age-related chronic conditions, as there are no well-established disease-modifying therapies for their prevention and treatment. Spatio-temporal distribution of tau protein pathology correlates with cognitive decline and severity of the disease, therefore, tau protein has become an appealing target for therapy. Current knowledge of the pathological effects and significance of specific species in the tau aggregation pathway is incomplete although more and more structural and mechanistic insights are being gained using biophysical techniques. Here, we review the application of NMR to structural studies of various tau forms that appear in its aggregation process, focusing on results obtained from solid-state NMR. Furthermore, we discuss implications from these studies and their prospective contribution to the development of new tauopathy therapies.

## Introduction

Neurodegenerative tauopathies form a large group of heterogeneous incurable diseases characterised by deposits of abnormal forms of tau protein in specific parts of the brain (Kovacs., 2015). The most frequent tauopathy is Alzheimer’s disease (AD). The number of patients with AD is estimated to be more than 30 million worldwide and is expected to increase dramatically (World Health Organisation, 2020). AD and other tauopathies lead to severe personality changes, decline of thinking skills and loss of patients’ ability to carry out everyday tasks, leaving them fully dependent on medical care until death, which usually occurs 5–10 years after the clinical diagnosis (Reitz et al., 2011). The socioeconomic burden of the disease is enormous due to the protracted disease course and dependence on care of AD patients (Wimo et al., 2010).

Hitherto efforts to develop a therapy for AD or tauopathies in general have had an extremely low success rate in comparison with other chronic conditions (Cummings et al., 2014). Only one new AD treatment (Aducanumab) has been approved since 2003 despite hundreds of clinical trials in the last two decades (Cavazzoni, 2021). Aducanumab is a passive immunotherapy which can remove aggregated forms of amyloid-beta peptide, one of the two hallmarks of AD (Sevigny et al., 2016); however, its ability to slow cognitive decline of patients was not ascertained yet. Other potential therapies in clinical research focus on the protein tau, which forms intracellular neurofibrillary tangles in neurons and is ubiquitous in tauopathies. The tau protein neurofibrillary pathology may appear early in the pre-symptomatic phase of disease and its spatio-temporal distribution correlates with cognitive decline and disease severity (Braak and Braak, 1991; Braak et al., 2011). For these reasons, tau has become an appealing target for various therapeutic strategies including small-molecule inhibition of tau aggregation and phosphorylation, anti-sense oligonucleotide therapy, passive and active immunotherapy (Li and Götz, 2017; Novak et al., 2018b; Congdon and Sigurdsson, 2018; Jadhav et al., 2019). Particularly, the recently completed Phase 2 clinical trial of AADvac1, an active immunotherapy targeting tau mid-region indicated that therapy may potentially slow cognitive decline in a subgroup of patients with ascertained tau pathology (Novak et al., 2021).

**FIGURE 1:**
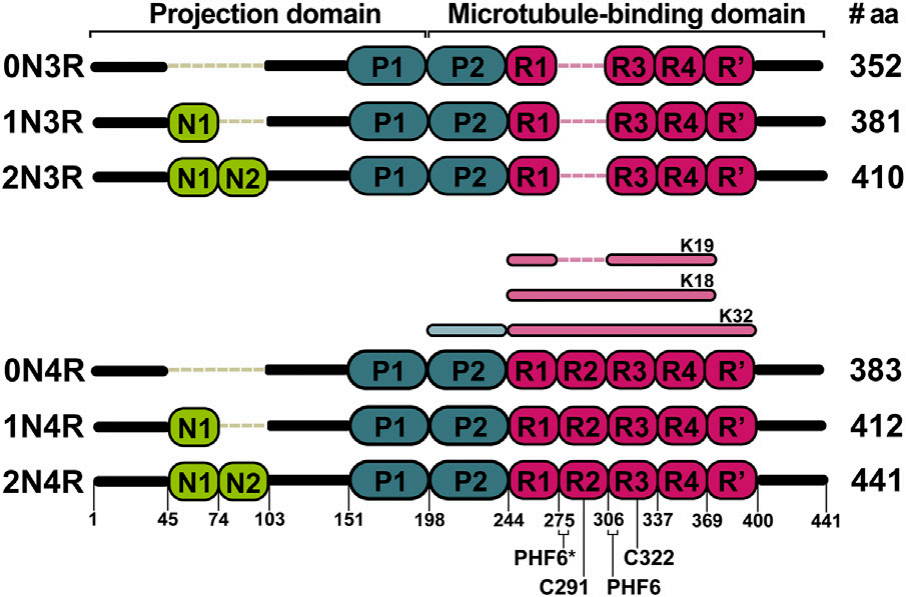
Six major tau isoforms generated by alternative splicing of the MAPT gene. N1 and N2 indicate N-terminal inserts (green). The presence of inserts is encoded by exon 2 and 3. P1 and P2 indicate proline-rich domains (blue). R1 to R4 indicate repeat domains (pink). The R2 is encoded by exon 10. The K18, K19, and K32 represent the most studied truncated constructs of tau both by solution and solid-state NMR. Locations of PHF6*, PHF6, C291, and C322 are shown on the 2N4R isoform.

In the brain, tau is expressed as six different isoforms comprising 352–441 residues. The isoforms are generated via alternative splicing of the MAPT gene and contain either zero, one, or two 29-residue inserts at the N-terminal part (0, 1 or 2N isoforms, respectively) and three to four repeat sequences at the C-terminal part (3R or 4R isoforms) (Figure 1). Compared to some other fibril-forming proteins, tau is a very soluble protein due to its high content of charged and hydrophilic amino acids (Sibille et al., 2006). Structurally, tau belongs to the class of intrinsically disordered proteins (IDPs), which do not form a stable tertiary fold or secondary structure elements and exist as an ensemble of interconverting conformations (conformational ensemble). Nevertheless, tau has many binding sites that are specific for different partners (Melkova et al., 2019). The protein can additionally undergo a large variety of posttranslational modifications, mostly phosphorylations, glycations and truncations, which modulate its physiological and pathophysiological functions (Zilka et al., 2012). The primary function of tau is to promote assembly and maintain stability of axonal microtubules, however, it has also been implicated in cellular signaling and regulation of other cellular processes (Habchi et al., 2014; Sotiropoulos et al., 2017). Self-assembly of tau is associated with tauopathies. Under normal conditions, tau has a low propensity to form aggregates. However, upon hyperphosphorylation (Alonso et al., 2001), metal ion binding (Jiji et al., 2017; Ahmadi et al., 2019) or truncation (Al-Hilaly et al., 2017; Novak et al., 2018a) tau self-assembles into insoluble paired helical (PHF) or straight filaments (SF), which can contribute to the pathogenesis. Repeat sequences present in the microtubule binding domain of all six isoforms are involved in the filament assembly (Goedert et al., 1988). Thus, the formation of filaments is linked to a reduced ability to bind microtubules.

A major bottleneck for development of new anti-tau therapies is the identification of the most relevant biological target (isoform, posttranslational modification, and aggregation state) to tackle. Understanding the structural and dynamic basis of tau assembly leading to disease is therefore crucial for developing new strategies for the treatment of AD and other tauopathies. The structure of tau filaments from AD patient-isolated material has been recently elucidated by cryo-EM (Fitzpatrick et al., 2017; Falcon et al., 2018). However, the filament structures fail to explain what is the exact basis for tauopathy-specific and structurally different filaments (Zhang et al., 2019). This points to a necessity for integrative structural and mechanistic studies addressing the interplay between truncation, phosphorylation, and aggregation of tau with respect to progression of pathology. NMR spectroscopy is unique in this sense as it can provide structural and functional information on tau’s disordered conformational ensembles, aggregated and filamentous states as well as directly probe phosphorylation and its effects.

In this paper, we review solution and solid-state NMR structural and interaction studies of tau in monomeric, oligomeric and filamentous forms. We start by reviewing the wide efforts to characterize secondary structure propensities of monomeric tau by solution NMR, and how it is influenced by phosphorylation or interaction with different partners. The following section describes the few studies of tau aggregation intermediates including oligomers. The last section is devoted to tau filament studies using solid-state NMR, which includes most recent results on *in vitro* tau fibrils generated without any inducer. Finally, we discuss the implications and perspectives of NMR studies to decipher the complex mechanisms of tau aggregation.

## Monomeric State as the Starting Point of Tau Self-Assembly

Until now, it has been unclear whether aggregation or phosphorylation is the leading event in the process of tau self-assembly into filaments (Lippens et al., 2003; Wegmann et al., 2021). However, for both of these events, the initial object of interest is a protein in the monomeric state. Protein aggregation is driven by a transition from α-helix or random coil to β-sheet structures (von Bergen et al., 2005; Ding et al., 2003; Bibow et al., 2011). Therefore, it is crucial to identify sites in proteins with a propensity to form β-strands, polyproline II helices or other extended structures amenable to self-association (Sillen et al., 2005a).

On the other hand, it is also necessary to explore whether any phosphorylation site may trigger the aggregation of tau. Hence, solution NMR studies of tau in the monomeric state have been performed with the aim to determine secondary structure propensities of the different tau protein regions and to measure the effects of phosphorylation at specific sites (Mukrasch et al., 2009; Smet et al., 2004; Bibow et al., 2011; Sibille et al., 2012).

### NMR Assignment

Assignment of backbone resonances is the first step towards any site-specific studies by NMR. However, a complete assignment of the backbone resonances of tau monomer in solution has been a major bottleneck due to its large molecular size and IDP character. Therefore, initial studies were performed on short peptides comprising fragments of the repeat sequences. Lippens and colleagues were among the first who tried to assign full-length tau. Their motivation was to use the assignment to study the impact of phosphorylation on tau. Unfortunately, using conventional assignment schemes, they were able to assign only a limited set of resonances (Lippens et al., 2003; Smet et al., 2004). Protein size, particular amino acid composition, repetitive regions, all together cause an immense overlap of signals, which makes assignment complicated (Lippens et al., 2006). The following attempts using higher magnetic fields and a 3D heteronuclear experiment setup were more successful, and Mukrasch and co-workers, whose primary motivation was to study structural propensities of tau and its interactions with microtubules and polyanions, presented the backbone assignment (except prolines) of full-length tau (Mukrasch et al., 2009). Later, other groups also succeeded in providing full-length tau assignments by implementing various non-uniform sampling (NUS)-NMR strategies allowing for higher dimensionality experiments (typically 4-7D) (Narayanan et al., 2010; Harbison et al., 2012) and analysis based on comparison with shorter constructs (Harbison et al., 2012). Some of these NUS-NMR strategies also succeeded to assign proline residues, in contrast to the conventional NMR assignment approaches. This is of particular importance for determination of the preferred trans/cis conformational states of individual prolines within tau in non-phosphorylated and a variety of phosphorylated states (Ahuja et al., 2016).

### Secondary Structure Propensities

In contrast to other methods such as CD spectroscopy which describe the overall populations of secondary structure elements within the studied protein, solution NMR allows to determine secondary structure propensities (SSP) at the individual residue level by mostly using the chemical shifts of Hα, Cα and Cβ atoms and their differences with respect to random coil values (Marsch et al., 2006).

**FIGURE 2:**
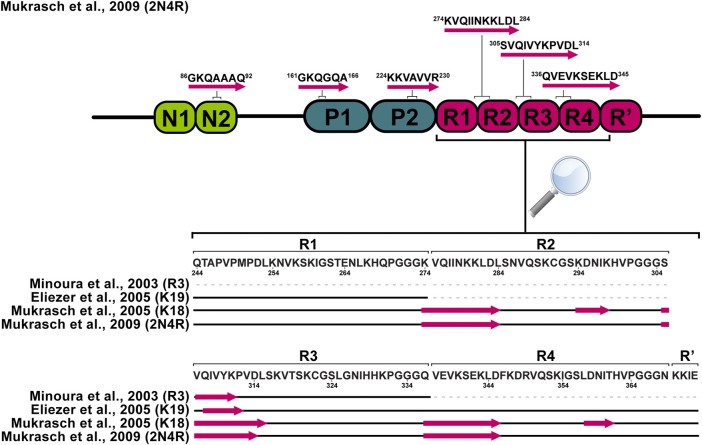
Schematic representation of tau monomer regions with preference for the β-sheet structure derived from solution NMR data. Pink arrows represent regions with increased β-sheet propensity.

More than two decades ago, von Bergen and colleagues reported that V306-K311 sequence (PHF6) in the R3 repeat (Figure 1) is a minimal tau self-interaction motif supporting the formation of filaments (von Bergen et al., 2000). This observation served as a motivation to study tau R3 by ^1^H NMR. Analysis of NOESY cross-peak pattern and ^3^ *J*_HNHα_ coupling data in 2,2,2-trifluoroethanol (TFE) suggested that the hexapeptide V306-K311 exhibits an extended structure, L315-L325 exhibits α-helix character, while the remaining part of the repeat remains unstructured. In addition, a possible model for the self-assembly via the helical structure was proposed, where the dimer formation and aggregation are promoted by hydrophilic and hydrophobic interactions, respectively. Contrary to previous findings, the sequence L315-L325 in H_2_O did not exhibit α-helix, but L315-S320 adopted an extended structure as V306-K311 (Minoura et al., 2002; Minoura et al., 2003). The possible reason for such a difference could be the tendency of TFE to stabilize α-helices (Shiraki et al., 1995). Unlike the R3, other repeats (R1, R2, and R4) in TFE do not exhibit any β-sheet propensity (Minoura et al., 2004; Tomoo et al., 2005). Deviations of Cα chemical shifts, which serve as sensitive probes for identifying local secondary structures, also identified a 6-residue region Q307-P312 with a β-sheet propensity in the cysteine-free K19 construct, which lacks R2 (Figure 2). (Eliezer et al., 2005). Mukrasch and colleagues expanded their interest and studied the K18 construct in addition to K19. Their data suggested that the beginning of each repeat except R1 has a stretch of 10–11 residues with a high β-structure propensity (Figure 2). The highest propensity was observed for K274-L284 and S305-L315, enclosing PHF6* and PHF6 hexapeptides (Mukrasch et al., 2005), which have been identified as seeds of filament formation (von Bergen et al., 2000). Moreover, a weak propensity for β-structures was observed in the ends of repeats R2 and R4 (Mukrasch et al., 2005). A later study on full-length tau supported the findings mentioned above (Figure 2). Additionally, several stretches with polyproline II helix propensity in the proline-rich regions P1 and P2 were observed, and a random coil character for 343 of 441 residues was confirmed (Mukrasch et al., 2009). Detailed comparison of SSP along the chain of tau and its homologue Map2c protein was also reviewed by Melkova et al. (Melkova et al., 2019).

### Phosphorylation Effects

**FIGURE 3:**
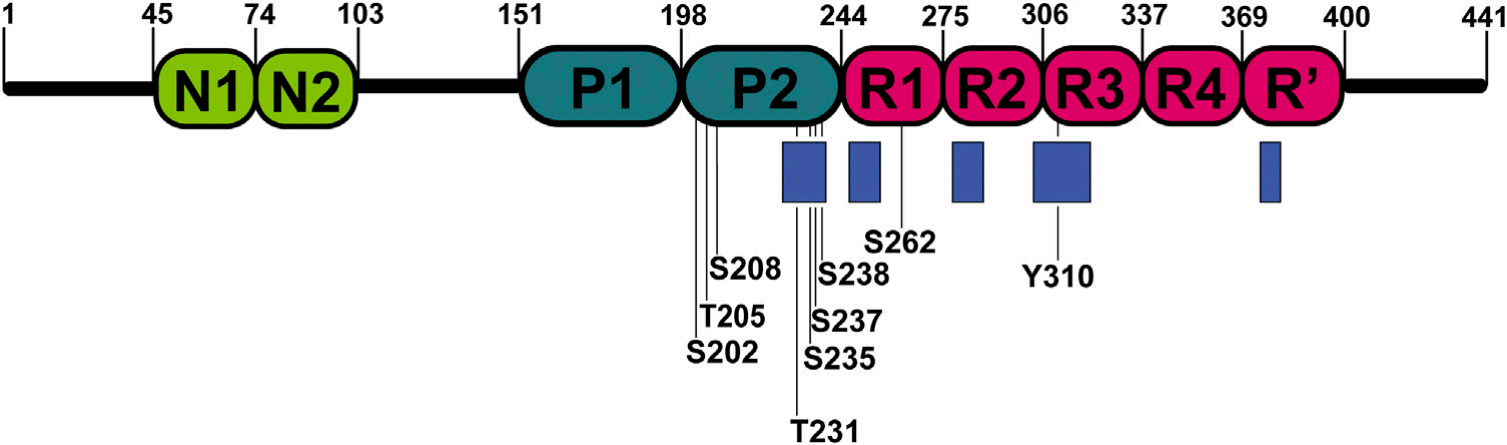
Locations of phosphorylated residues and tau microtubule-binding sites discussed in this review. Blue rectangles represent tau microtubule-binding sites with the strongest affinity.

Hyperphosphorylation has been suggested to be sufficient for the induction of tau filament formation (Alonso et al., 2001; Despres et al., 2019). Phosphate groups have the potential to impact the local and global structure of tau and alter its interaction preferences from microtubules to itself. Serines, threonines, and tyrosines, which are potential sites for phosphorylation, comprise nearly one-fifth of tau residues making it an ideal substrate for kinases (Lippens et al., 2003). Over the last two decades NMR has been used to decipher the pattern of tau phosphorylation in both qualitative and quantitative manner (Lippens et al., 2003; Landrieu et al., 2006; Amniai et al., 2009; Amniai et al., 2011; Qi et al., 2016; Despres et al., 2017). The NMR studies were focused on the phosphorylation of residues in the proline-rich domains and repeat regions of the protein (Figure 3). Solution NMR has also allowed monitoring the phosphorylation kinetics of multiple residues by multiple kinases. Recent advances in the use of non-uniformly sampled NMR have offered superior time resolution of such processes (Mayzel et al., 2014; Louša et al., 2017). This resulted not only in the efficient comparison of different phosphorylation rates at several sites but also allowed to determine whether any phosphorylation is pre-conditioned by a previous phosphorylation at a different site.

According to Hα chemical shift deviations, in a short peptide (residues K224-K240) from the proline-rich domain double phosphorylation at T231 and S235 induced β-turn propensity for residues V229–T231 (Daly et al., 2000), while multiple-site phosphorylation induced a structural change to polyproline II helix in engineered proline-rich domain peptides. Moreover, the data derived from ^*3*^ *J*_*HαHN*_ suggested that these changes may be involved in the global structural transition of phosphorylated tau to the aggregated form (Bielska and Zondlo, 2006). Likewise, an enhanced propensity for β-turn formation of V229–T231 was also observed in a case where phosphorylation was mimicked by mutations T231E and S235E. In addition, a transient helix between S238 and R242 was found, which does not depend on phosphorylation of T231 but is stabilized by phosphorylation of S235, S237, and S238 (Sibille et al., 2012; Schwalbe et al., 2015). Based on NOE and molecular dynamics (MD) data, a turn conformation was hypothesised as a response to phosphorylation at S202 and T205. The turn is stabilized by a hydrogen bond between the phosphorylated T205 and the amide proton of G207 (Gandhi et al., 2015). The phosphorylation of only S202 and T205 has been suggested to be protective against aggregation. If G209, which forms the stabilizing hydrogen bond, is mutated to valine and combined with phosphorylation at S202, T205, and S208, tau forms filaments without any other aggregation inducer (Despres et al., 2017).

The impact of phosphorylation in the repeat region of tau has been also studied. In the R1 peptide (residues V256-G273), phosphorylation of S262 enhanced the assembly rate of the peptide in comparison to the non-phosphorylated version. The different assembly rates can be explained by differences in conformation between R1 and phospho-R1 (pR1), derived from chemical shift perturbations of HN and Hα between R1 and pR1 (Zhou et al., 2006). In contrast, other studies suggest that phosphorylation of S262 inhibits aggregation of tau (Schneider et al., 1999; Haj-Yahya et al., 2020). The same site was studied in the K18 construct (Figure 1); instead of introducing genuine phosphate groups, the phosphorylation effects were mimicked by mutating serine to glutamic acid in the tau repeats. The mutation did not significantly influence the secondary structure propensities of repeats. Although, mutations induced selective conformational changes in the R1 and R2 (Fischer et al., 2009). Phosphorylation of another site Y310 in the R3, associated with the formation of PHFs, was sufficient to delay tau aggregation (Ait-Bouziad et al., 2020). A possible explanation could be that the interaction of I308 and non-phosphorylated Y310 is crucial for forming tau filaments (Naruto et al., 2010; Sogawa et al., 2014).

### Interaction Studies

The primary function of tau is to regulate essential functions of microtubules (MTs), including polymerization, stabilization, and modulation of dynamics. Therefore, details about tau binding to microtubules are highly important for understanding these functions (Mukrasch et al., 2005). The reduced affinity of tau to MTs is mediated by genetic mutations or hyperphosphorylation and leads to axonal transport perturbation. When it occurs, tau detaches from the MT complex and tends to aggregate (Kadavath et al., 2015a). On the other hand, specific tau truncation may result in formation of abnormal forms of tau-MT complexes (Novak et al., 2018a). Tau is also able to interact with many other partners, including several molecules that have been implicated in pathogenesis, such as polyanions, metal ions and other amyloid proteins. Hub proteins, for example 14-3-3 proteins, which connect multiple cellular pathways should also be considered. These interactions have been extensively studied using solution NMR.

The overlay of 2D ^1^H-^15^N HSQC spectra of free and MT-bound tauF4 (residues S208-S324) revealed the disappearance of a large fraction of the resonances, which was caused by slow tumbling in solution upon binding to the microtubules (Sillen et al., 2007; Kadavath et al., 2015a). The most affected residues were K224–S237, T245–L253, V275–L284, and V300–K317, comprising P2, R1, R2, and R3 parts of the protein. Results were nearly identical for both 3R and 4R tau isoforms, indicating that they share the same attachment mechanism to the MTs (Kadavath et al., 2015a). Chemical shift perturbations measured from 2D ^1^H-^15^N HSQC spectra highlighted positively charged lysine and histidine residues in the repeat sequences preceding the PGGG motifs as mediators of the microtubule binding (Mukrasch et al., 2005). The most significant chemical shift changes, pointing to a strong involvement in the MT-binding process, were observed for residues K225-T231 in P2 domain, K240-V248 in R1 domain, V275-S285 and I297-V300 in R2, and K370-K375 in R’ (Mukrasch et al., 2007). Other studies have suggested that FTDP-17-associated mutations (∆K280 and P301L), phosphorylation of S214, and pseudophosphorylation of KXGS motifs significantly attenuate the binding to microtubules (Fischer et al., 2007; Sillen et al., 2007; Fischer et al., 2009). These results are in agreement with the “jaws” model of tau binding whereby the regions flanking the repeats are considered as targeting domains, responsible for positioning and high affinity tau binding on the MTs surface, and the repeats act as catalytic domains for microtubule assembly (Fischer et al., 2007; Kadavath et al., 2018). The results are also in agreement with atomic models of MT-bound tau derived from a combination of cryo-EM data and Rosetta modeling (Kellogg et al., 2018), which indicate that tau attaches to MTs through repeat sequences. However, slightly different residues in R1 were found to be involved in the binding in comparison to NMR results (Kadavath et al., 2015b).

In-cell NMR has over the years evolved into a well-established, valuable tool to study proteins in close-to-native conditions (Serber and Dötsch, 2001; Montheith and Pielak., 2014; Theillet et al., 2016; Luchinat and Banci., 2018). Until now, only a single study has been published on in-cell NMR experiments of tau protein, focusing on a shorter fragment (Zhang et al., 2018). In this work, the authors acquired 2D HSQC spectra of the ^15^N-labelled K19 fragment in several buffer conditions and subsequently, upon electroporating the protein into HEK-293 cells. The spectrum of K19 under *in situ* conditions was most similar to the one where K19 was incubated *in vitro* with polymerized MTs, indicating that tau may primarily interact with MTs *in situ*. The authors also highlighted that the most significant change in signal intensity was observed in the PHF6 region (V306-K311). Additionally, a MARK2-phosphorylated version of K19 in the HEK-293 cells was observed to undergo rapid dephosphorylation of residues S262, S324, S352, S356 shortly after introduction into the cells. The spectrum of full-length tau showed widespread chemical shift perturbations, with most significant changes around the PHF6 region, similarly to K19, and with signals of V309, Y310, and K311 broadened beyond detection. Finally, immunofluorescence-based co-localization with tubulin in SH-SY5Y cells pointed to tau interacting with MTs, and NMR suggested the involvement of the PHF region in this interaction *in vivo*. These experiments highlight the potential of in-cell NMR to examine structural changes of tau in near-native conditions at atomistic resolution (Zhang et al., 2018).

In most cases, tau aggregation *in vitro* is initiated by polyanions, such as heparin, polyglutamic acid, and RNA (Mukrasch et al., 2005). Several polyanions were also found in brain-derived tissues of AD patients (Goedert et al., 1996; Paudel and Li., 1999). Upon polyanion binding tau protein’s highly positive net charge is partially shielded, which facilitates formation of β-sheet structures. The polyanions directly stabilize the regions essential for aggregation (Mukrasch et al., 2005; Akoury et al., 2016). NMR experiments suggested that tau binds polyanions via the same interaction sites as MTs. Thus, the most noticeable changes of backbone amide chemical shifts were similarly observed in proximity of lysine and histidine residues (Mukrasch et al., 2005; Sibille et al., 2006; Fischer et al., 2007; Mukrasch et al., 2007). Moreover, NMR revealed that the polyanion binding increases residual β-sheet propensity within R2 and R3 hexapeptides, which were identified as the seeds of tau filament formation (Sibille et al., 2006; Akoury et al., 2016). Taken together, phosphorylation and polyanions diminish tau interaction with microtubules by blocking the interaction sites and adapt them for the formation of filaments (Mukrasch et al., 2005).

Metal ions are essential for normal brain function. However, during AD pathogenesis these ions accumulate in the brain of patients (Ahmadi et al., 2019). *In vitro* studies revealed that metals like Zn^2+^ and Cu^2+^ bind to tau and increase its aggregation rate (Jiji et al., 2017). The mode of metal-binding to tau was investigated by NMR titration. The binding site of Cu^2+^ was located in repeats R2 and R3, in particular, V287-S293 and Y310-S324. Moreover, H299 in R2, H329 and H330 in R3 contributed to the interaction (Soragni et al., 2008). Another study proposed an alternative interaction mode of Cu^2+^ coordinated to H268 in R1 and H363 in R4, which promoted the dimerization of R2 and R3 via C291-C322 disulfide bond formation (Ahmadi et al., 2019). In the case of Zn^2+^, its binding was mapped to the R3 repeat, particularly C322, and histidines H268, H329/H330, were found to complement the cysteine in Zn^2+^ binding (Jiji et al., 2017). These studies show a slightly different interaction mechanism between Zn^2+^ and Cu^2+^, although in both cases the histidines and cysteines present in the repeat sequences participate in the coordination of these ions. Such interactions may result in stabilization of a particular conformation, particularly β-turn structures.

Another important connection can be found between tau, phosphorylation and the 14-3-3 protein family. The 14-3-3 protein isoforms are highly expressed in the human brain and interact with thousands of protein partners (Sluchanko and Bustos, 2019; Gogl et al., 2021). It has been proposed that 14-3-3 proteins might be in a competitive relationship with tubulin for binding to tau (Hashiguchi et al., 2000; Qureshi et al., 2013; Chen et al., 2019). 14-3-3 proteins have been found colocalised with tau in NFTs extracted from AD brains (Layfield et al., 1996; Umahara et al., 2004), and they were suggested to promote tau protein aggregation and fibrillization in a phosphorylation-dependent manner (Hernández et al., 2004; Sadik et al., 2009). As described above, tau’s interaction with microtubules is heavily influenced by its phosphorylation status (Lindwall and Cole, 1984; Biernat et al., 1993). For example, phosphorylation by PKA is known to decrease tau-tubulin binding, by modifying S214, T231 or S356 (Scott et al., 1993; Scheider et al., 1999). At the same time, it generates binding epitopes for the 14-3-3 proteins (Sluchanko et al., 2009; Joo et al., 2015). This may seriously affect microtubule stability and cell viability. Indeed, *in vivo* experiments have shown significantly retarded axonal development in neuronal cultures overexpressing 14-3-3, via microtubule destabilization (Joo et al., 2015; Li and Paudel, 2016). The precise role of 14-3-3 in the process of tau aggregation remains to be elucidated, however, NMR studies have highlighted their interaction sites.

Using chemical shift perturbation mapping, the binding region(s) of 14-3-3 on phosphorylated full-length tau have been described in detail (Joo et al., 2015; Andrei et al., 2018). High quality ^1^H-^15^N HSQC spectra demonstrated a significant signal reduction of residues throughout the MTBR and proline-rich regions of tau (mainly in the vicinities of S214 and S324), while resonances in the projection and C-terminal domains were nearly unaffected. Notably, this description is strikingly similar to the regions responsible for tubulin binding (Sillen et al., 2007).

Tau has been shown to form soluble complexes with amyloid beta that may promote their aggregation into the insoluble forms observed in AD (Guo et al., 2006; Jin et al., 2011). Similarly, the presence of monomeric α-synuclein was found to promote formation of tau co-aggregated fibrils (Lu et al., 2020; Hojjatian et al., 2021). Therefore, cross-interactions of tau with other proteins involved in neurodegenerative diseases (α-synuclein and Aβ40) have been studied by NMR. NMR chemical shift perturbations revealed that α-synuclein interacts mainly with the PHF6 motif of tau through its negatively charged C-terminal region (Lu et al., 2020). α-Synuclein fibrils formed in the presence of tau were recently characterized by ssNMR experiments revealing that they share similar conformation with one particular type of fibrils obtained in the absence of tau (Hojjatian et al., 2021). In contrast, addition of full-length tau did not induce chemical shift perturbations in the ^1^H-^15^N HSQC spectrum of ^15^N labeled Aβ40, although small losses in signal intensity were observed immediately after addition of tau pointing to weak interaction. As a result of this, the co-incubation of Aβ and tau induced amorphous aggregates and inhibited Aβ40 from fibrillization (Wallin et al., 2018). These examples show that tau cross-interactions also need to be considered in its aggregation mechanism.

## Oligomers and Other Intermediates as a Black Box of Tau Aggregation

In spite of all gained knowledge of the potential triggers of tau aggregation, the mechanism of how soluble tau undergoes assembly into insoluble filaments is not well explored. Intermediates in this process are oligomers. Over the last decades, several studies have shown that toxic soluble oligomers could initiate the neurodegeneration cascade. Moreover, there is rising evidence that the onset of Alzheimer’s disease and other tauopathies occurs earlier than tau filaments are found in the brain. Therefore, it is crucial to characterize intermediates along the aggregation pathway (Berger et al., 2007; de Calignon et al., 2010; Lasagna-Revees et al., 2012; Ghag et al., 2018), which may be the most relevant form to target with anti-tau therapies.

**FIGURE 4:**
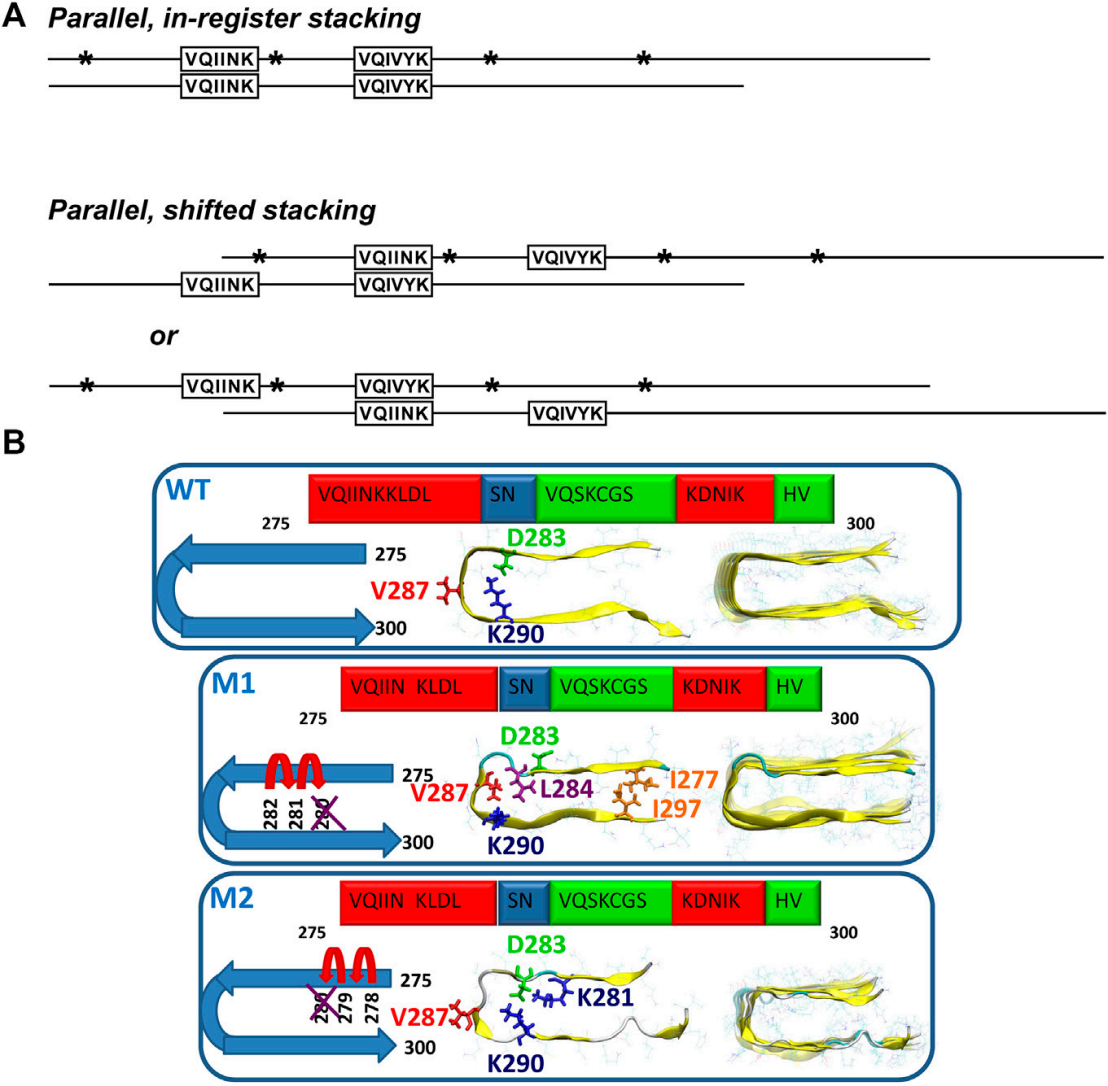
**(A)** Models of possible tau oligomer complexes generated during heparin-induced aggregation (parallel, in-register stacking, and parallel, shifted stacking). Asterisks show the positions of single MTSL derivatizations in relation to the regions observed to be broadened: ^275^V-K^280^ and ^306^V-K^311^. Adapted with permission from Peterson et al. (2008). Copyright 2008 American Chemical Society. **(B)** Schematic representation of two oligomer structural models of the mutated ΔK280 tau repeat R2: models M1 and M2. In M1, the deletion mutation was obtained by ‘shifting’ the C-terminal sequence towards K280. In M2, the deletion mutation was obtained by ‘shifting’ the N-terminal sequence towards K280. Reproduced from Raz et al. (2014) with permission from the PCCP Owner Societies.

The contribution of NMR to the characterization of oligomers remains scarce. Soluble oligomers of tau187 (residues N255-L441), which comprises all four repeats and the C-terminal domain of tau, were detected by solution NMR. Backbone resonances of monomeric tau187 were assigned using conventional 3D triple-resonance experiments. Further, paramagnetic relaxation enhancements (PREs) were measured in response to heparin-induced aggregation of ^15^N labeled tau, which highlighted two MTSL-broadened regions, V275-K280 and V306-K311. In line with this observation, it was proposed that soluble oligomers are generated via parallel, in-register and parallel, shifted-register intermolecular interactions at these regions (Figure 4A). However, it remained unclear whether these oligomer species belong to the on-pathway oligomers (Peterson et al., 2008). PRE NMR was also used to capture the soluble oligomers of tau in response to interaction with the organic compound pthalocyanine tetrasulfonate (PcTS), which inhibits tau aggregation. Moreover, the study results indicated that the formed off-pathway oligomers are structurally distinct from toxic oligomers of tau (Akoury et al., 2013).

The ΔK280 deletion mutant, which is known to accelerate tau aggregation and is associated with the development of frontotemporal dementia (Wegmann et al., 2011) was studied by a combination of solid-state NMR, atomic force microscopy, transmission electron microscopy (TEM) and all-atom explicit molecular dynamics simulations in the R2 peptide (Raz et al., 2014). The authors found that this deletion mutation induces the formation of oligomers and reduces the generation of fibrils. Two structural models of the oligomers were proposed by ‘shifting’ the sequence from the C- or the N-terminal end towards the ΔK280 mutation site (Figure 4B). Model M1 is characterized by mostly hydrophobic contacts, whereas in the model M2, the dominant interactions are salt bridges. The ssNMR chemical shift assignment of R2 in ΔK280 revealed that labeled residues D283, V287, and K290 are in β-sheet conformation. In the simulated model M1, V286 and K290 show the β-sheet conformation, while, in the M2 model, D283 and V287 show the β-sheet conformation. The molecular dynamics data illustrated that M1 adopts a relatively well-packed structure compared with the M2 model. Comparison of the relative conformational energies and the populations of models shows that model M1 is more stable and strongly preferred over model M2. Therefore, the authors proposed that larger populations of the self-assembled ΔK280 tau R2 repeat oligomers and fibrils are organized as in the model M1 (Raz et al., 2014).

## Filaments as Aggregation End-Products

Until the mid-1990s, studies of tau filaments were limited to patient-derived material due to the unavailability of well-established methods to spontaneously aggregate tau protein *in vitro* (Despres et al., 2019). Since the breakthrough discovery by Goedert et al. that heparin, a polyanionic cofactor, can trigger the formation of filaments of non-phosphorylated tau protein, aggregation of tau *in vitro* has been done by its addition. Heparin screens electrostatic interactions, which result in conformational rearrangement of tau protein, leading to its self-assembly (Goedert et al., 1996; Fichou et al., 2018). Besides heparin, other polyanionic compounds such as heparan sulfate (Zhao et al., 2020), RNA, arachidonic acid (Sibille et al., 2006), polyglutamic acid (Akoury et al., 2016) can be used for spontaneous aggregation of tau. It has also been reported that metal (Cu^2+^, Zn^2+^) ions can trigger the aggregation process (Soragni et al., 2008; Jiji et al., 2017; Ahmadi et al., 2019). This variety of inducers demonstrates that tau aggregation is rather influenced by electrostatics than by the specific interactions with the inducer (Sibille et al., 2006).

Recent cryo-EM progress showed that heparin-induced tau filaments are structurally heterogeneous and distinct from those in Alzheimer’s and Pick’s disease, questioning the relevance of such aggregation protocols (Fichou et al., 2018; Zhang et al., 2019). For this reason, more recently, methods to obtain filaments without an aggregation inducer are used in the tau scientific community and latest NMR studies. Usually, truncated constructs of tau comprising only the repeat sequences are used in such an approach. For example, tauF4 (residues S208-S324) (Huvent et al., 2014), dGAE (residues I297-E391) (Al-Hilaly et al., 2019), R3R4 (residues V306-F378) (Carlomagno et al., 2021; Jayan et al., 2021) are able to form filaments without addition of an inducer reagent. Moreover, Carlomagno et al. showed that R3R4 can serve as a seed and promote the aggregation of full-length tau (Carlomagno et al., 2021).

### Heparin-Induced Aggregates

In electron micrographs, a rigid core of tau filaments appears to be surrounded by a fuzzy outer coat (Wischik et al., 1988; Sillen et al., 2005b). Protein regions, which are incorporated in the core of the filaments of tau, are broadened beyond the detection limits of solution NMR due to immobilization, while residues in the outer coat outside the core retain a significant degree of mobility and should be observable by solution NMR (Sillen et al., 2005a; Sillen et al., 2005b). The first attempt to study heparin-induced tau filaments by ssNMR was made by Sillen and coworkers (Sillen et al., 2005b). The authors analysed the intensity of backbone amide peaks in the ^1^H-^15^N HSQC spectrum as a function of primary sequence location, which allowed identification of protein regions with distinct mobility after assembly into PHFs. At the flexible N-terminal part of the protein, full intensity was recovered for the amide peaks up to A77. The following residues up to the proline-rich region showed a linear reduction of peak intensity. Most of the proline-rich region (residues T205-R230) displayed a residual intensity ratio below 30% and was defined as semi-rigid with complex dynamics. The rigid PHF core was mapped to the region G261-T386 with the lowest signal intensities. The C-terminal residue peak intensities were partially recovered indicating that the rigid core does not extend to the C terminus of tau (Sillen et al., 2005a). Later PRE measurements were used to probe long-range interactions of the rigid core and the fuzzy coat of tau filaments. Nitroxide spin label attached to C322 caused signal broadening in the first 30 residues of the N-terminus as well as in residue stretches close to Q124, A152, N167–T212, and S409-A426 at the C-terminal part (Bibow et al., 2011).

**FIGURE 5:**
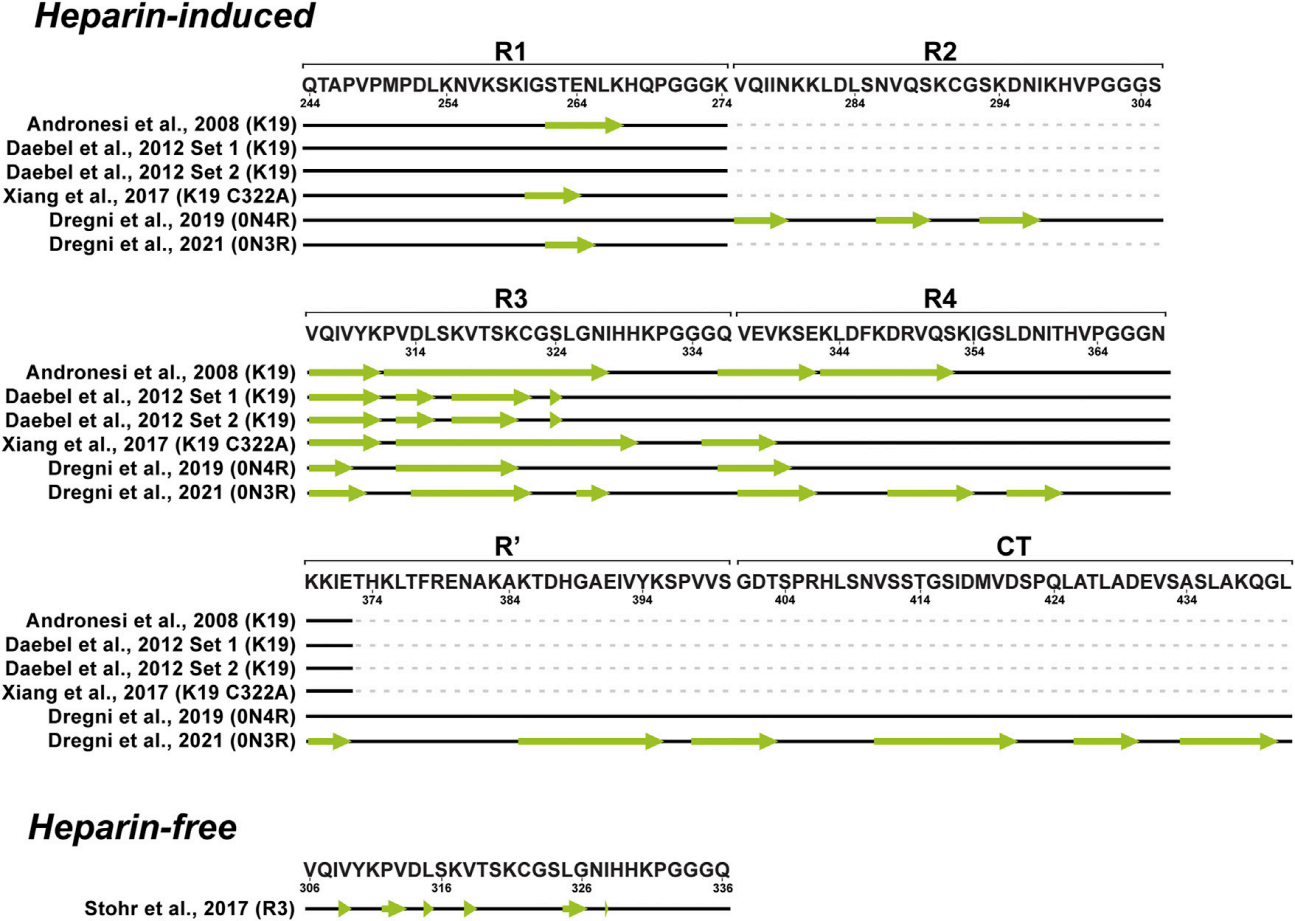
Schematic representation of β-strand locations (indicated with green arrows) in fibrillar tau derived from solid-state NMR data.

Tau variants intensively studied by ssNMR were the truncated constructs K18, K19 and K32 (Figure 1) encompassing the core of native PHFs. In the case of heparin-fibrilized filaments, the K19 is the most studied construct of tau. Despite high sample heterogeneity causing considerable line broadening in ssNMR spectra, a complete resonance assignment was obtained for 43 residues in the rigid parts of the construct and 29 residues in the mobile N- and C-terminal part (Andronesi et al., 2008). Secondary chemical shifts were derived based on NCA and NCOCA data sets, which indicated strong β-sheet character for several residues in R1, R4, and the entire R3 as manifested by largely negative secondary chemical shift values, and random-coil or α-helical conformation near the N-and C- termini. The exact locations of β-strands were determined by analysing the chemical shifts together with correlations observed in an hNhhC experiment. These data indicated a short β-strand at the end of R1 (βR1, residues S262–K267), two β-strands in R3 (residues Q307–I328), and two β-strands in R4 (residues Q336–I354) (Figure 5). Moreover, the H_2_O-edited NCA experiment suggested that βR1 and βR4 strands are more solvent-exposed in comparison to βR3 strand, which is more likely to be buried in the filament core. Lack of long-range and intermolecular contacts hampered the generation of a structural model, however, the authors proposed the relative arrangement of molecules in filaments. The burial of the βR3 strand suggested that it may form the interface within the minimal structural unit of K19 filaments comprising two molecules connected via a disulfide bridge (Andronesi et al., 2008).

In the following study Daebel and coworkers compared the dynamics of the repetitive regions (Daebel et al., 2012). Unique residues in each repeat, A246, Y310 and F346 were used as sentinel residues for R1, R3 and R4, respectively. Chemical shifts assigned using an INEPT-CC-TOBSY spectrum probed a random coil character for A246 and F346, while Y310 resonance was absent. This indicated that R1 and R4 are much more flexible compared to R3. Additionally, the role of the R2 repeat in the rigid core was investigated using filaments obtained from the K18 construct, which showed an overall behavior similar to K19. The R2 repeat was found to be protected from the exchange with solvent, similarly to R3. In contrast to Andronesi et al., about 80% of K19 resonances were not observed, possibly, due to increased heterogeneity. Nevertheless, PDSD experiments at various temperatures revealed that most residues become rigid at sub-zero temperatures. Using a selectively labeled sample, the oxidation state of the single cysteine residue (C322) in the K19 sequence was investigated. Two cysteine resonances were detected, which both exhibited oxidized cysteine chemical shifts, suggesting the presence of at least two conformations (Figure 5). Upon cysteine mutation to alanine, a simplified resonance pattern was observed, thereby confirming the role of the cysteine in producing structural heterogeneity (Daebel et al., 2012).

The K19 C322A mutant was studied by Xiang and colleagues. ssNMR spectra and electron micrographs showed noticeable heterogeneity with several types of filaments within the sample although the same aggregation protocol as before was used implying batch-to-batch variability (Xiang et al., 2017; Daebel et al., 2012; Andronesi et al., 2008). Despite this heterogeneity, the authors employed proton-detected experiments with up to four chemical shift dimensions resulting in extensive chemical shift assignments for residues G260-E264 and V306-K340 (Figure 5). The authors concluded that tau filaments inherently exist as an ensemble of structures with a consistent and well defined structure only in the hexapeptide motif (Xiang et al., 2017).

On the other hand, Savastano and coworkers studied the involvement of the P2 region in filaments using the K32 construct that spans residues S198-S400 (Savastano et al., 2020). Regrettably, the resulting PDSD spectrum displayed signal overlap, giving just a few isolated peaks. However, the comparison of K32 and K19 PDSD spectra suggested that the R1 and the R3 repeats are part of the rigid cross-β structure in the K32 filaments. Evidence of P2 involvement in the rigid core structures was provided by ssNMR analysis of the model peptides P2R2 and P2R3. Upon aggregation, the resonances of P2 domain peptide K225-T231, which resembles the hexapeptides in repeats R2 and R3, lost their intensities compared to the monomeric state of the peptides.

**FIGURE 6:**
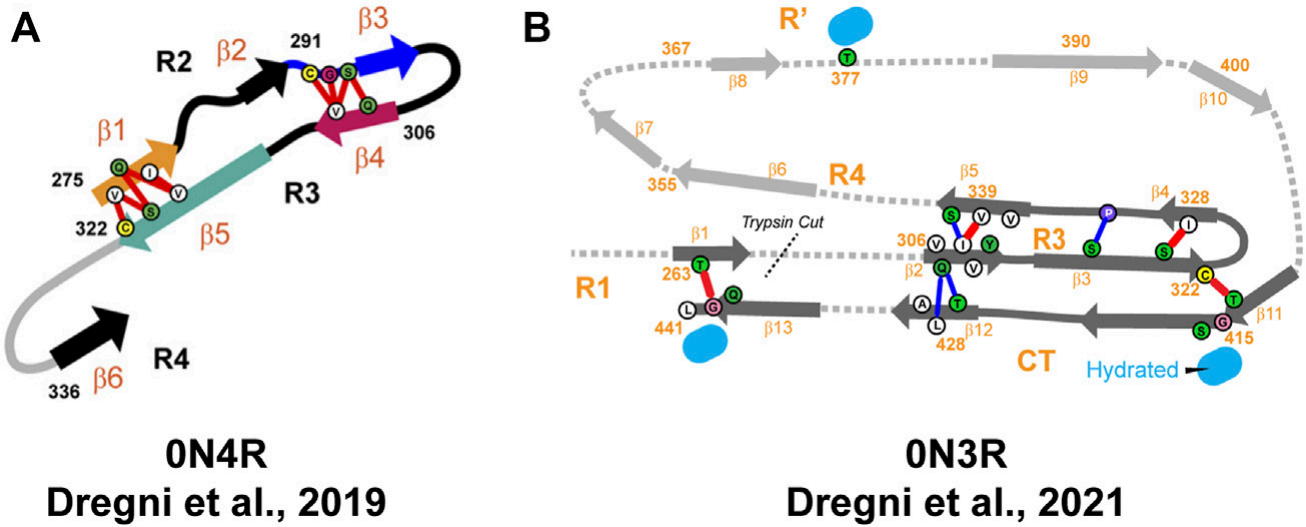
**(A)** Structural model of the heparin-fibrillized 0N4R tau core. Schematic arrangement of the β-strands (thick arrows) and long-range correlations (red lines) measured in the ssNMR spectra. Hypothetical locations of segments outside the R2–R3 core are shown as gray lines. Orange, blue, magenta, and green arrows highlight the crucial R2 hexapeptide motif, the C291-containing segment, the R3 hexapeptide motif, and the C322-containing segment. Adapted with permission from Dregni et al. (2019). Copyright 2019 National Academy of Sciences. **(B)** Structural model of the heparin-fibrillized 0N3R tau. Unambiguous long-range contacts are shown as red lines while ambiguous contacts are shown as blue lines. The 3D fold of residues 342–411 is indicated by gray arrows and dotted lines. Adapted with permission from Dregni et al. (2021). Copyright 2021 American Chemical Society.

An ssNMR structural model of 0N4R tau filaments has been published by Dregni et al. (Figure 6) (Dregni et al., 2019). For the first time, homogeneous filaments yielding high-quality ssNMR data were obtained. Assignment was performed for residues G270-K340 and the chemical shift–derived torsion angles indicated six β-strands (β1–β6) starting from the R2 hexapeptide motif ^275^VQIINK^280^ and ending with the ^336^QVEVK^340^ segment at the beginning of R4 (Figure 5). Remarkably, the assigned ^13^C and ^15^N chemical shifts of the 0N4R construct differed significantly from the truncated K18 and K19 tau constructs as well as showed different locations of β-strands (Andronesi et al., 2008; Daebel et al., 2012; Xiang et al., 2017). Therefore, the fibril core of 0N4R tau is distinct from tau filaments studied previously. 2D CC and 3D NCACX correlation spectra with a long mixing by ^13^C–^13^C CORD (Hou et al., 2013) spin diffusion were used to determine the overall fold of the 0N4R fibril core. Cross-peaks indicating close proximities were observed between the β3 strand (in R2) and the β4 strand (hexapeptide motif in R3), β1 strand and β5 strand as well as the β3 and β4 strands. In the model, the rigid β-sheet core spans residues V275-Q336 and is shaped like a hairpin (Figure 6), with β1 and β5 marking the approximate beginning and end. The intermolecular packing corresponds to parallel-in-register. The authors also noted the presence of a semi-rigid β-sheet domain flanking the filament core (Dregni et al., 2019).

The previous structural model of 0N4R fibrils included only R2 and R3 parts of the protein sequence. In order to characterize the other parts in 0N4R, NMR relaxation and hydration studies were performed (Dregni et al., 2020). The authors concluded that the exterior of a β-sheet hairpin formed by R2-R3 is well-protected from water by other residues. Interestingly, the less rigid R1 and R4 domains did not exhibit significant differences in water accessibility compared to R2 and R3 domains, indicating their limited exposure to water molecules. Water-edited 2D^13^C–^13^C and ^15^N-^13^C correlation spectra showed that S285 and S316 are the most hydrated residues in the β-sheet core, while other serine, threonine and cysteine residues are poorly hydrated. S285 and S316 face each other in a central pocket, which led to the conclusion that the interior of the R2-R3 hairpin contains a small water pore. In addition, this water pore is local because the water-edited S285 signal is significantly more intense than that of the neighboring S289. The poor hydration of the 0N4R tau fibril core and small size of the water pore suggest that semi-rigid R1 and R4 domains or flanking regions outside repeat sequences are better targets of small-molecule drugs and imaging agents than the R2-R3 region. Although the core of the 0N4R fibril model does not include the R4 and R′ domains, the authors spotted similarities with the corticobasal degeneration CBD tau structure comprising the R2-R′ domains (Zhang et al., 2020). Both models exhibit the R2-R3 hairpin and a significant kink between the R2 hexapeptide and S285. Additionally, the hydration and dynamics data suggested that R4 is rigid and participates in hydrogen bonding in some units, while R3 is protected from water. The authors concluded that R3 and R4 are packed together like in the CBD tau fold and increased dynamics of R4 and R’ is in agreement with lower resolution in the cryo-EM structure (Dregni et al., 2020).

Recently, the same group published another ssNMR derived structural model of homogeneous 0N3R filaments (Figure 6) (Dregni et al., 2021). A comparison of 2D ^1^H−^15^N correlation INEPT spectra between 0N3R and 0N4R highlighted missing peaks in the 0N3R spectrum corresponding to residues at the C-terminus, which indicated that the C-terminal domain is not isotropically mobile in the 0N3R isoform in contrast to 0N4R. Furthermore, a larger number of alanine peaks in the NCA spectrum of 0N3R were observed, which is consistent with the inclusion of the C-terminus into the β-sheet core since part of R′ and the C-terminus include seven alanines. The site-specific backbone assignments of the 0N3R tau fibril core were obtained for 104 out of 149 residues covering the region S262-L441 confirming the rigidity of the C-terminal residues. The peak intensities of R3, R4, R′, and C-terminal residues were comparable in dipolar correlation spectra, whereas the R1 signals were less intense, consistent with the start of the rigid core from the middle of R1. The proposed model showed that the 0N3R isoform has six β-strands in similar locations to 0N4R (G261-S262 in R1, V306−Y310 and V313−H330 in R3, V337−S341, V350−K353, and N359-T361 in R4) and four additional β-strands K385−S400, V411−S422, A426-A429, and A434-G440 in R′ and C-terminal part (Figure 5). The model was created based on 90 medium-range contacts obtained from spectra with long spin diffusion times, which included four unambiguous and four ambiguous long-range contacts that could not be explained by any short-range contacts. These eight long-range contacts provided explicit constraints on the tertiary fold of the 0N3R tau core indicating that the R1-R3 stretch is packed against the C-terminal region in an antiparallel fashion and that the R3 packs against the R4 repeat. In this filament model, the core has an elongated C-shape resembling an alligator head (Figure 6), which differs qualitatively from all *in vivo* and *in vitro* tau fibril core structures known to date (Dregni et al., 2021).

### Inducer-free Spontaneously Formed Aggregates

The observed polymorphism and doubts about the biological relevance of heparin-induced filaments have recently inspired a surge in the studies of spontaneously aggregated tau. Stohr and colleagues studied a set of different peptides derived from the R3 domain of tau (Stöhr et al., 2017). For ssNMR analysis, filaments of full-length R3 peptide formed under reducing conditions (R3_SH_) were used, chosen for its high biological activity and macroscopic homogeneity confirmed by TEM and fibre diffraction. Two peptides with specific labeling schemes were synthesized for the magic angle spinning (MAS) ssNMR studies, and 2D^13^C–^13^C DARR correlation spectra acquired at 12 kHz MAS were used for assignment. Single peaks with narrow ^13^C line widths were observed for all labelled residues except L315. Secondary chemical shift analysis indicated that all labelled residues, except L315 and G326, exhibit β-sheet conformation (Figure 5). For L315, the major conformer was predicted to be in the β-sheet conformation, while a minor conformer was in a non-beta conformation. The authors hypothesized that the proximity of P312 could break the β-sheet conformation. The ssNMR data were in excellent agreement with HDX experiments, which showed increased mobility in proximity to P312. The authors concluded that the R3 peptides are a small but biologically relevant system for addressing detailed biophysical questions regarding tau filament formation. Moreover, the full R3 repeat could serve as a template for full-length tau aggregation into filaments (Stöhr et al., 2017).

Previously, it has been shown that short peptides from tau repeat regions can self-assemble and form fibrils, and that spontaneous aggregation is less efficient as the length of tau fragment increases (Schweers et al., 1995; Stohr et al., 2017). Jayan and coworkers investigated the aggregation of R3R4 tau. In order to confirm the presence of R4 repeat in the fibril core, fibrils formed *in vitro* without heparin were studied using ssNMR (Jayan et al., 2021). Lack of NMR peaks in the INEPT-CC-TOBSY experiments suggested the absence of flexible residues in the R3R4 fibrils. The ^13^C–^13^C PDSD experiments showed slightly increased line widths of the peaks suggesting that there was still structural polymorphism in the fibrils. Secondary chemical shift analysis showed that all assigned residues in the R3 have β-strand conformation. The PDSD spectra also allowed to assign 13 amino acids of the R4 repeat, which was concluded to be a part of the rigid core, in line with the various fibril structure models from cryo-EM (Fitzpatrick et al., 2017; Falcon et al., 2018). Thus, this study suggests that the R3R4 is a suitable model system for *in vivo* tau filaments (Jayan et al., 2021).

**FIGURE 7:**
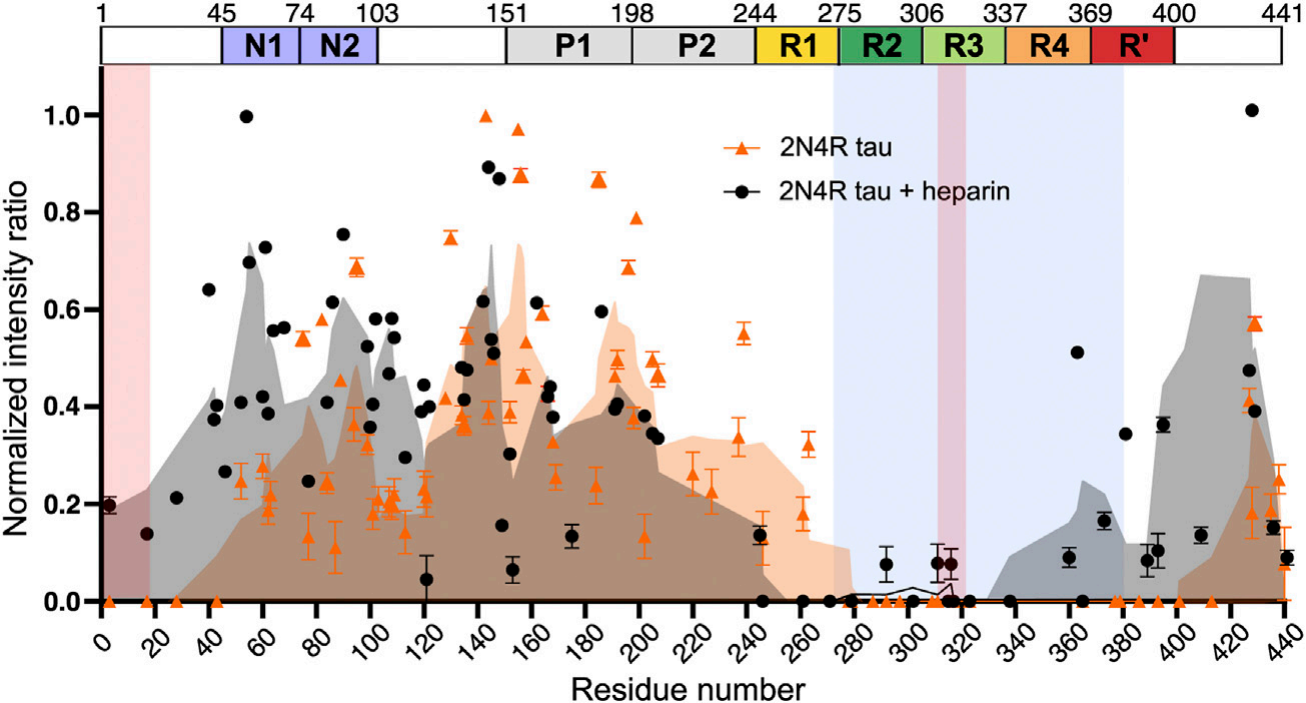
Intensity ratio plot of INEPT signal of 2N4R tau fibrils aggregated in the absence (orange) and presence (black) of heparin. The intensity ratio was calculated by dividing the signal intensity of each residue in the fibril state by the monomeric state. The rigid cross-ß-sheet core of the tau fibril extracted from a CBD patient brain (PDB code: 6TJO) is marked in light blue. Adapted with permission from Chakraborty et al. (2021). Copyright 2021 Nature Publishing Group.

Recently, 2N4R isoform filaments prepared in the absence of heparin were studied by ssNMR (Chakraborty et al., 2021). The analysis of ^1^H-^15^N INEPT spectra, which detects only highly dynamic residues in solid samples, revealed a complete loss of signals from residues P270-S400 (Figure 7). This is in contrast to the case of heparin-fibrilized 2N4R, for which signals from residues I260-H330 were not detected in the INEPT spectrum (Figure 7). These data suggest that filaments generated in the absence of heparin have a similar fibril core length as CBD tau fibrils (Zhang et al., 2020). Interestingly, 30 residues at the N-terminus could also not be observed suggesting a transient interaction between fibril core and the N-terminus. The peak widths in the 2D RFDR and 2D NCA spectra of the heparin-free 2N4R tau fibrils indicated structural homogeneity of the rigid core. However, only a few residues in the fibril core could be assigned due to strong signal overlap. To gain further insight into the structural properties of heparin-free 2N4R tau fibrils, dynamic nuclear polarisation (DNP)-enhanced 2D hChhC and hNhhC spectra of selectively labeled (^13^Cγ valine, ^13^C-ring phenylalanine, ^15^N histidine) fibrils were measured. Two cross-peaks observed between the Cγ of valine and the ring carbons of phenylalanine suggested that the side chains of one or two valine residues are in proximity to the side chain of a phenylalanine residue. Another cross-peak between the Nε1/Nδ2 atoms of histidine side chain and Cγ of valine indicated that these groups are in ∼4 Å distance in the structure of 2N4R fibrils. These contacts are in agreement with the cryo-EM structure of CBD fibrils, which shows that F346 is in proximity of V350, F378 contacts V275 and aromatic ring of H330 is close to Cγ of V318 (Zhang et al., 2020).

## Conclusion

Solution and solid-state NMR have allowed bridging the gaps in structural knowledge of tau in the monomeric, oligomeric, and filamentous states. In comparison to other structural methods, solution NMR has provided residue specific insights into tau monomer regions with transient secondary structures, phosphorylation patterns and interactions with binding partners. Thus, the hexapeptide sequence motifs present in R2 and R3 repeats have been identified by several studies as regions with highest (up to 25%) β-sheet propensity (or extended conformation). Phosphorylation using site-specific kinases has been found to increase the β-sheet propensity in proline-rich regions, which could result in reduced tau binding to microtubules. The effect of polyanions is similar to phosphorylation as they were also shown to increase residual β-sheet propensity within R2 and R3 hexapeptides, thereby facilitating filament assembly. However, solution NMR studies on tau were primarily devoted to examining the role of repeats while regions outside the filament-forming core have not been characterized to the same extent. Phosphorylation studies have remained scarce and have mostly addressed isolated sites, therefore effects of hyperphosphorylation should be studied in the future, potentially using other kinases and employing also ^31^P NMR. Additionally, it would be important to apply NMR for characterization of tau interactions with synthetic compounds or peptides that stabilize the monomeric state, thereby contributing to development of new AD therapies.

The weakest link in the chain of tau research has been oligomer characterization despite their high biological relevance and potential toxicity. NMR studies of tau oligomers have been hampered mainly because of limited capabilities to prepare stabilized aggregation intermediates. Soluble oligomers obtained by heparin-induced aggregation were characterized using PRE-NMR in solution, which indicated that the hexapeptides V275-K280 and V306-K311 participate in parallel, in-register and parallel, shifted-register intermolecular interactions. However, it remains unclear whether the oligomers detected in this way are on the aggregation pathway. The ΔK280 deletion mutant of the R2 repeat, which favors formation of oligomers over fibrils, was studied by solid-state NMR. The authors proposed a structural model of the ΔK280 oligomers, which comprised two β-strands (V275-L284 and K290-V300) assembled in a hairpin structure. Further studies of tau aggregation intermediates are necessary to elucidate the aggregation pathway and understand the disease-specificity of filament folds. The recent discovery of shorter tau constructs, which can be aggregated spontaneously, may lead to new approaches for studying oligomers by solution and solid-state NMR. In particular, sample freezing in solid-state DNP-NMR experiments could be used to stabilize and detect low-populated aggregation intermediates with sufficient sensitivity. Additionally, solution NMR could be used to monitor monomer depletion and oligomer formation during tau aggregation in a site-specific manner using selectively labeled samples.

NMR has played a major role in the initial structural characterization of tau filaments, obtained by heparin-induced aggregation. The rigid PHF core was mapped to the repeat region (residues G261-T386), whereas the proline-rich region (residues T205-R230) was defined as semi-rigid with complex dynamics. A major bottleneck for filament structure determination has been structural heterogeneity, and some studies have even concluded that tau filaments inherently exist as an ensemble of structures with a consistent and well-defined structure only in the hexapeptide motifs. To reduce the sample heterogeneity due to transient long-range interactions, solid-state NMR experiments were performed with the truncated tau variants K18, K19, K32 and/or cysteine mutants, since it was determined to play a role in producing structural heterogeneity. This has allowed determination of locations of secondary structures, their involvement in the rigid core and relative protection from solvent for K19, K18 and K32 filaments. The first structural model of heparin-induced tau filaments was determined for the 0N4R construct owing to preparation of homogeneous filaments. The model comprised six β-strands covering the protein region from R2 hexapeptide to the beginning of R4, which only partially overlapped with the locations of β-strands in K18 and K19 filaments. The overall fold of the 0N4R fibril resembled a hairpin and the tau proteins had parallel-in-register intermolecular packing. Recently, another structural model of homogeneous tau 0N3R filaments was published. A major difference with respect to the 0N4R filaments was the inclusion of the C-terminus in the β-sheet core. In the model, which has an elongated C-shape, six β-strands show similar locations to 0N4R and four additional β-strands are located in the R′ and C-terminal parts. However, the heparin-induced tau filament models differ qualitatively from the structures of all patient-derived tau filaments determined by cryo-EM. Therefore, several recent studies have been performed with shorter constructs including full-length R3 peptide and R3R4 that aggregate spontaneously. Such constructs have been suggested as suitable model systems for *in vivo* tau filaments and could also serve as templates for aggregation of full-length tau. Another example is the solid-state NMR study of 2N4R isoform filaments prepared in the absence of heparin, which revealed a similar fibril core length and long-range contacts consistent with CBD patient-derived tau fibrils studied by cryo-EM. These recent results suggest that ssNMR should move towards studies of spontaneously formed aggregates as well as aggregates formed by seeding with shorter constructs or patient-derived material. Although cryo-EM has recently made significant progress in the structural characterization of patient-derived tau filaments, there are still several fundamental questions unanswered (e.g., what exactly determines the different tau filament structural signatures in individual human tauopathies). Recent technological advances such as proton detection at very fast MAS and DNP-NMR give additional confidence that some of those questions could be answered using NMR.

In conclusion, detailed understanding of the effects and significance of individual changes in the tau assembly pathway is required for selecting the best molecular species to target with new AD therapies. In this review, NMR studies of various monomeric, oligomeric and filamentous species in solution and in solids have been considered, which together allow the sketching of a plausible aggregation pathway. Nevertheless, further research is required, particularly to characterize on-pathway intermediate aggregates, which remain a black box in the mechanism of tau aggregation. Also, the interplay between tau truncation and phosphorylation in relation to aggregation behavior and the final filament structure as well as co-aggregation with (cross)-interaction partners are unaddressed problems. Many of the underlying questions are well suited for NMR, thus, we can expect significant contributions in the tau field from NMR studies in the future.
